# Formal Caregivers in Intellectual Disability Facilities

**DOI:** 10.1192/j.eurpsy.2023.1890

**Published:** 2023-07-19

**Authors:** B. Martins, I. A. Silva, J. Cardão, A. H. de Matos, C. Agostinho

**Affiliations:** Psychiatry and Mental Health Department, Unidade Local de Saúde do Norte Alentejano, Portalegre, Portugal

## Abstract

**Introduction:**

Caring for individuals with intellectual disabilities is a passionate, but challenging profession. Whether working in residential homes or in occupational facilities, its staff deals with the ordinary issues of teamwork, but also with these clients behavioral comorbidities. It often includes self-harming and aggression towards others – namely towards the staff itself. This is particularly relevant since a high turnover of staff implies the loss of people who are familiar with the needs and specificities of those clients. The quality of care provided may also be affected.

**Objectives:**

To investigate whether, or not, formal caregivers are at greater risk of occupational health issues, and what their main determinants are.

**Methods:**

Research in Medline for *intellectual disability caregivers.* Only the relevant articles, published in English, were considered.

**Results:**

Among formal caregivers of people with intellectual disabilities, job dissatisfaction and job strain were found to be especially relevant and were associated to the following variables:

• Younger workers or those with less professional experience

• Personality and individual maladaptive coping strategies

• Poor organizational support

• Conflicting, ambiguous, or overloaded professional roles

• Unclear work tasks

The incidence of Burnout Syndrome was also described as relevant among these workers and was associated not only to aggression towards the worker, but also to the fear of aggression.

**Conclusions:**

Caring for people with intellectual disabilities, in a residential or in an occupational context, implies heavy emotional and physical demands and a high prevalence of job dissatisfaction, Job Strain and Burnout Syndrome. In addition to the high risks to the quality of life and health of caregivers, there are consequences for the quality of the care that is provided. For this reason, guaranteeing proper work conditions should be considered part of the Social Institutions main goals. Yet, we lack specific, controlled studies that properly evaluate what measures could indeed help institutions to prevent occupational (and mental) health distress among their staff.

**Disclosure of Interest:**

None Declared

